# Antibacterial and phytochemical screening of *Anethum graveolens*, *Foeniculum vulgare *and *Trachyspermum ammi*

**DOI:** 10.1186/1472-6882-9-30

**Published:** 2009-08-06

**Authors:** Gurinder J Kaur, Daljit S Arora

**Affiliations:** 1 Microbial Technology Laboratory, Department of Microbiology, Guru Nanak Dev University, Amritsar-143005, Punjab, India

## Abstract

**Background:**

*Anethum graveolens *Linn., *Foeniculum vulgare *Mill. and *Trachyspermum ammi *L. are widely used traditional medicinal plants to treat various ailments. To provide a scientific basis to traditional uses of these plants, their aqueous and organic seed extracts, as well as isolated phytoconstituents were evaluated for their antibacterial potential.

**Methods:**

Antibacterial activity of aqueous and organic seed extracts was assessed using agar diffusion assay, minimum inhibitory concentration and viable cell count studies; and their antibacterial effect was compared with some standard antibiotics. The presence of major phytoconstituents was detected qualitatively and quantitatively. The isolated phytoconstituents were subjected to disc diffusion assay to ascertain their antibacterial effect.

**Results:**

Hot water and acetone seed extracts showed considerably good antibacterial activity against all the bacteria except *Klebsiella pneumoniae *and one strain of *Pseudomonas aeruginosa*. Minimum inhibitory concentration for aqueous and acetone seed extracts ranged from 20–80 mg/ml and 5–15 mg/ml respectively. Viable cell count studies revealed the bactericidal nature of the seed extracts. Statistical analysis proved the better/equal efficacy of some of these seed extracts as compared to standard antibiotics. Phytochemical analysis showed the presence of 2.80 – 4.23% alkaloids, 8.58 – 15.06% flavonoids, 19.71 – 27.77% tannins, 0.55–0.70% saponins and cardiac glycosides.

**Conclusion:**

Antibacterial efficacy shown by these plants provides a scientific basis and thus, validates their traditional uses as homemade remedies. Isolation and purification of different phytochemicals may further yield significant antibacterial agents.

## Background

Nature has served as a rich repository of medicinal plants for thousands of years and an impressive number of modern drugs have been isolated from natural sources, notably of plant origin [1]. Herbal medicine, based on their traditional uses in the form of powders, liquids or mixtures, has been the basis of treatment for various ailments in India since ancient times.

The use of herbs as complementary and alternative medicine has increased dramatically in the last 20–25 years [2]. According to World Health Organization (WHO) traditional medicines are relied upon by 65–80% of the World's population for their primary health care needs. Moreover, emergence of multiple drug resistant strains of microorganisms due to indiscriminate use of antibiotics to treat infectious diseases has generated a renewed interest in herbal medicine [3]. The beneficial health effects of many plants, used for centuries as seasoning agents in food and beverages, have been claimed for preventing food deterioration and as antimicrobials against pathogenic microorganisms. Antimicrobial potential of different medicinal plants is being extensively studied all over the world [4-7] but only a few studies have been carried out in a systematic manner. However, in the absence of any scientific proof of their effectiveness, the validity of these remedies remains questionable and their use locally restricted. Phytochemical and pharmacological investigations of several plants have already led to the isolation of some of the natural antimicrobials [8].

In the present study, three medicinal plants *viz. Anethum graveolens *Linn. (Dill), *Foeniculum vulgare *Mill. (Fennel) and *Trachyspermum ammi *L. (Omum) belonging to the family Umbelliferae were selected to assess their antibacterial potential. These plants are a common household remedy against a variety of gastrointestinal disorders, e.g. indigestion, flatulence, colic pain etc.; also used as spices and condiments in foods for their flavour, aroma, and preservation; and their dried ripe fruits and essential oils have aromatic, carminative, stomachic and diuretic properties [9]. The essential oils of these plants have been reported to possess antimicrobial activity [10-14]. However, in folklore, seeds or their aqueous extracts are used as homemade remedies but only a little work has been done to explore them. To the best of our knowledge, the present study is the first positive report using aqueous extract of seeds in contrast to an earlier study [4]. Thus, to provide a scientific justification for these traditional remedies, the present study was planned to assess their antibacterial potential using aqueous and organic extracts against some clinically important bacteria. Phytochemical screening was carried out to identify major biologically active phytoconstituents.

## Methods

### Materials

All the chemicals and standard antibiotics were purchased from Hi-Media, Mumbai, India; and al the solvents used were of analytical grade. Precoated silica gel 60 F_254 _TLC plates and standard phytoconstituents were purchased from Merck, Germany and Sigma Chemicals, USA, respectively.

### Bacterial cultures

Reference bacterial strains *viz. Enterococcus faecalis *(MTCC 439), *Staphylococcus aureus *(MTCC 96), *Escherichia coli *(MTCC 119), *Klebsiella pneumoniae *1 (MTCC 109), *K. pneumoniae *2 (MTCC 530), *Pseudomonas aeruginosa *1 (MTCC 647), *P. aeruginosa *2 (MTCC 741), *Salmonella typhi *(MTCC 531), *Salmonella typhimurium *1 (MTCC 98), *S. typhimurium *2 (MTCC 1251) and *Shigella flexneri *(MTCC 1457) were obtained from Microbial Type Culture Collection (MTCC), Institute of Microbial Technology (IMTECH), Chandigarh. These were maintained on nutrient agar slants except *Enterococcus faecalis *which was maintained on trypticase soya agar (TSA). All the isolates were sub cultured regularly and stored at 4°C as well as at -80°C by making their suspension in 10% glycerol.

### Inoculum preparation

A loopful of isolated colonies was inoculated into 4 ml of peptone water, incubated at 37°C for 4 h. This actively growing bacterial suspension was then adjusted with peptone water so as to obtain a turbidity visually comparable to that of 0.5 McFarland standard prepared by mixing 0.5 ml of 1.75% (w/v) barium chloride dihydrate (BaCl_2_. 2H_2_O) with 99.5 ml of 1% (v/v) sulphuric acid (H_2_SO_4_). This turbidity is equivalent to approximately 1–2 × 10^8 ^colony forming units per ml (CFU/ml).

### Plant materials

Seeds of different plants *viz. Anethum graveolens*, *Foeniculum vulgare *and *Trachyspermum ammi *were obtained from the local market of Amritsar. All the plant materials have been deposited vide accession number 6419 (V, VI and VII) in Herbarium, Department of Botanical and Environmental Sciences, Guru Nanak Dev University, Amritsar, Punjab, India.

### Preparation of seed extracts

Aqueous/organic extracts of seeds were prepared by taking the weighed amount of each sample in known volume of water/organic solvent to get the desired concentration (200 mg/ml). Seeds of different plants were surface sterilized using 1% mercuric chloride (HgCl_2_) and crushed using pestle and mortar. Aqueous extracts of seeds were prepared in three different ways as described earlier [15]. Organic extracts of seeds were prepared using four different solvents with increasing polarity-hexane, ethyl acetate, acetone, and ethanol. Weighed amount of each sample was extracted in known volume of the solvent for 24 h with intermittent shaking. Each extracted material was filtered through Whatman filter paper No. 1 and centrifuged at × 10 000 g for 15 min and the supernatant was used for antibacterial testing.

### Effect of grinding

To assess the effect of grinding on antibacterial activity, the seeds were shade dried; powdered using electric blender and their aqueous and organic extracts were prepared using the same methods as described above.

### Determination of antibacterial activity by agar diffusion method

Sensitivity of different bacterial strains to various extracts was measured in terms of zone of inhibition using agar diffusion assay (ADA) [16]. The plates containing Mueller-Hinton/Nutrient agar were spread with 0.2 ml of the inoculum. Wells (8 mm diameter) were cut out from agar plates using a sterilized stainless steel borer and filled with 0.1 ml of the extract. The plates inoculated with different bacteria were incubated at 37°C up to 48 h and diameter of any resultant zone of inhibition was measured. For each combination of extract and the bacterial strain, the experiment was performed in duplicate and repeated thrice. The bacteria with a clear zone of inhibition of more than 12 mm were considered to be sensitive. The antibacterial activity of different plant extracts was compared with eight commonly employed antibiotics *viz*. ampicillin (10 μg/disc), cefixime (5 μg/disc), chloramphenicol (30 μg/disc), co-trimoxazole (25 μg/disc), gentamicin (10 μg/disc), imipenem (10 μg/disc), pipericillin/tazobactam (10 μg/disc) and tobramycin (10 μg/disc).

### Minimum inhibitory concentration (MIC)

Minimum inhibitory concentration of the effective seed extracts was worked out by agar dilution method [17]. Nutrient agar plates containing varying concentrations (10–100 mg/ml aqueous extract; 1–50 mg/ml acetone extract) of different seed extracts were prepared and inoculated with 0.1 ml of the inoculum. The plates were incubated at 37°C for 24 h and the lowest concentration of the extract causing complete inhibition of the bacterial growth was taken as MIC. The results were compared with that of control using sterilized distilled water/acetone. The experiment was performed in duplicate and repeated three times.

### Bactericidal activity

Bactericidal activity of hot water seed extracts prepared at 40°C at a concentration of 200 mg/ml was measured by viable cell count method [18]. Five ml of 4 h grown inoculum was serially diluted to 10^-3 ^with double strength nutrient broth. Equal volumes of the diluted inoculum and the extract to be tested were mixed and incubated at 37°C. At different time intervals *viz*. 0, 1, 2, 3 ..., 24 h, 0.1 ml of the mixed suspension was spread on two separate nutrient agar plates and incubated for 24 h at 37°C. The mean number of colonies were obtained and compared with that of control in which the seed extract was replaced with sterilized distilled water. Each experiment was repeated thrice. The results were expressed as number of viable cells as a percentage of control.

### Phytochemical screening

The powdered seeds were evaluated for qualitative and quantitative determination of major phytoconstituents *i.e. *alkaloids, flavonoids, tannins, saponins and cardiac glycosides; which were further confirmed by thin layer chromatography.

### Qualitative screening

Alkaloid detection was carried out by extracting 1 g powdered sample with 5 ml methanol and 5 ml of 2N HCl; and then treating the filtrate with Meyer's and Wagner's reagents. The samples were scored positive on the basis of turbidity or precipitation. Flavonoids were tested by heating 1 g powdered sample with 10 ml ethyl acetate over a steam bath (40–50°C) for 5 min; filtrate was treated with 1 ml dilute ammonia. A yellow colouration demonstrated positive test for flavonoids. The presence of tannins was confirmed by boiling 0.5 g powdered sample in 20 ml distilled water, followed by addition of 3 drops of 5% FeCl_3 _to the filtrate. Development of brownish-green or blue-black colouration was taken as positive for the presence of tannins. Saponins content was determined by boiling 1 g powdered sample in 10 ml distilled water for 15 min and after cooling, the extract was shaken vigorously to record froth formation. Cardiac glycosides were identified by extracting 2 g sample in 10 ml methanol. Five ml of this methanolic extract was treated with 2 ml glacial acetic acid containing 1 drop of 5% FeCl_3 _solution. This solution was carefully transferred to surface of 1 ml conc. H_2_SO_4_. The formation of reddish brown ring at the junction of two liquids was indicative of cardenolides/cardiac glycosides [19].

### Thin layer chromatography (TLC)

Identification of major phytoconstituents was further carried out by TLC using pre-coated silica gel 60 F_264 _plates [20]. Different screening systems were used to obtain better resolution of the components. Standard markers such as atropine, rutin, catechin, glycyrrhizic acid and lanatoside C were co-chromatographed for alkaloids, flavonoids, tannins, saponins and cardiac glycosides, respectively. The developed plates were observed under visible as well as UV light (254 nm and 356 nm). R_f _value of each spot was calculated as – R_f _= Distance travelled by the solute/Distance travelled by the solvent

### Bioautography of extracts

Qualitatively isolated group of compounds which were subjected to thin layer chromatography [20], were also assessed for their antibacterial potential using agar disc diffusion assay. Alkaloids were isolated by mixing 1 g powdered sample with 1 ml of 10% (v/v) ammonia solution and extracted with 5 ml methanol for 10 min on water bath (40°C). It was then filtered through Whatman filter paper No. 1 and the filtrate was concentrated using rotary evaporator. Isolation of flavonoids was achieved by heating 1 g powdered sample with 5 ml methanol on water bath at 40°C for 10 min. The filtrate was then concentrated using rotary evaporator to 1/4^th ^of its original volume. For saponins, one gram powdered sample was extracted with 5 ml methanol by heating on a water bath at 40°C for 10 min. The extract was filtered and evaporated to 1 ml, mixed with 0.5 ml water and then extracted thrice with 3 ml n-butanol. The n-butanol phase was evaporated and concentrated to approximately 1 ml. Tannins were obtained by treating 1 g powdered sample with 10 ml 2 M hydrochloric acid (HCl) and hydrolyzing in boiling water bath for 30 min. The solution was filtered, mixed thoroughly with 1 ml ethyl acetate, and ethyl acetate layer was then discarded. Five drops of amyl alcohol were added and shaken thoroughly. Alcoholic layer was retained and used for antibacterial testing. Cardiac glycosides were isolated by extracting 1 g powdered sample with 5 ml of 50% (v/v) methanol and 10 ml of 10% (w/v) lead (II) acetate solution by heating on water bath at 40°C for 10 min. The filtrate was cooled to room temperature and then extracted twice with 10 ml dichloromethane/isopropanol (3:2). The combined lower phases were filtered over anhydrous sodium sulphate and evaporated to dryness. The residue was dissolved in 1 ml dichloromethane/isopropanol (3:2) and this solution was further used for antibacterial investigations. Sterile discs (4.5 mm) cut out from Whatman filter paper No. 1 were saturated with all the five isolated group of compounds, air dried and used for antibacterial activity testing.

### Quantitative analysis

Alkaloids were quantitatively determined according to the method of Harborne [19]. Two hundred ml of 10% acetic acid in ethanol was added to 5 g powdered sample, covered and allowed to stand for 4 h. The filtrate was then concentrated on a water bath to 1/4^th ^of its original volume. Concentrated ammonium hydroxide was added drop wise to the extract until the precipitation was complete. The whole solution was allowed to settle; collected precipitates were washed with dilute ammonium hydroxide and then filtered. The residue was dried, weighed and expressed as the alkaloids.

To estimate flavonoids quantitatively, 10 g powdered sample of each plant material was extracted twice with 10 ml of 80% aqueous methanol at room temperature. The whole solution was filtered through Whatman filter paper No.1, the filtrate was later transferred into crucibles, evaporated to dryness on a water bath to a constant weight [21,22].

Quantitative determination of saponins was done according to Obadoni and Ochuko [23]. Twenty gram of each powdered sample was added to 100 ml of 20% aqueous ethanol and kept in a shaker for 30 min. The samples were heated over a water bath for 4 h at 55°C. The mixture was then filtered and the residue re-extracted with another 200 ml of 20% aqueous ethanol. The combined extracts were reduced to approximately 40 ml over water bath at 90°C. The concentrate was transferred into a 250 ml separatory funnel, extracted twice with 20 ml diethyl ether. Ether layer was discarded while aqueous layer was retained and 60 ml n-butanol was added to it. Then n-butanol extracts were washed twice with 10 ml of 5% aqueous sodium chloride. The remaining solution was heated in a water bath and after evaporation the samples were dried in oven (40°C) to a constant weight. The saponin content was calculated as percentage of the initial weight of sample taken.

Tannin determination was done according to the method of Van-Burden and Robinson [24] with some modifications. Distilled water (50 ml) was added to 500 mg of the sample taken in a 500 ml flask and kept in shaken for 1 h. It was filtered into a 50 ml volumetric flask and made up to the mark. Then 5 ml of the filtrate was pippetted out into a test tube and mixed with 2 ml (10 fold diluted) of 0.1 M FeCl_3 _in 0.1 N HCl and 0.008 M potassium ferrocyanide. The absorbance was measured at 605 nm within 10 min.

### Statistical analysis

All values have been expressed as mean ± standard deviation and the comparison of the antibacterial activity of the samples with standard antibiotics was evaluated by applying t-test. P ≤ 0.05 values were considered to indicate statistically significant difference.

## Results and discussion

The results of present study are encouraging as all the tested plants showed antibacterial potential, although the inhibitory activity was strain specific. The method of extracting the plant material and its form (whether crushed or finely powdered) affected their antibacterial activity. Powdered seed extracts showed considerable loss of antibacterial activity in comparison to extracts prepared by using crushed seeds, which could be attributed to inactivation of the active antibacterial substances by the heat generated during grinding of the seeds (using an electric blender).

### Antibacterial activity of aqueous extract of seeds

Out of the aqueous extracts prepared in three different ways, hot water extract of seeds (prepared at 40°C) gave better inhibition zones as compared to extracts prepared at ambient temperature of water and boiling water; which might be attributed to the incomplete leaching of the antibacterial substances at ambient temperature and the loss of the active ingredient/s during boiling. Thus, hot water extracts of the crushed seeds were selected for further experimentation.

The aqueous extracts of different plant seeds resulted in variable zone of inhibition (11–25 mm) for all the bacteria tested except *K. pneumoniae *1, 2 and *P. aeruginosa *1, which were completely resistant (Table 1). Hot water extracts of all the seeds were effective against *E. faecalis*, *S. aureus*, *E. coli*, *P. aeruginosa *2, *S. typhimurium *and *S. flexneri*; while *S. typhi *was sensitive only to aqueous extracts of *F. vulgare *(12 mm). In another study aqueous extracts of *F. vulgare *and *T. ammi *did not show any antibacterial activity [4].

**Table 1:** Antibacterial activity of aqueous extract of seeds

Bacteria	Extracts/Zone of inhibition (in mm)								
	P 1^a^			P 2^a^			P 3^a^		
	I^a^	II^a^	III^a^	I^a^	II^a^	III^a^	I^a^	II^a^	III^a^
EF	17	24	15	13	22	-	13	22	10
SA	23	25	18	22	24	-	22	23	12
EC	-	12	-	14	15	-	16	17	-
KP 1	-	-	-	-	-	-	-	-	-
KP 2	-	-	-	-	-	-	-	-	-
PA 1	-	-	-	-	-	-	-	-	-
PA 2	21	24	20	21	24	17	22	24	19
ST	-	-	-	11	12	-	-	-	-
STM 1	-	12	12	-	-	-	-	-	-
STM 2	13	20	-	14	14	-	11	12	-
SF	-	14	-	18	18	-	14	15	-
									
Mean	6.73	11.91	5.91	10.27	11.73	1.55	8.82	10.18	3.73
± SD	9.645	10.492	8.420	8.776	10.061	5.126	9.097	10.381	6.724

^a ^P1: *Anethum graveolens*; P2: *Foeniculum vulgare*; P3: *Trachyspermum ammi*

Aqueous extracts (I: Ambient water; II: Hot water; III: Boiling water extract)

-: no zone of inhibition

EF: *Enterococcus faecalis*; SA: *Staphylococcus aureus*; EC: *Escherichia coli*; KP 1: *Klebsiella pneumoniae *1; KP 2: *K. pneumoniae *2; PA 1: *Pseudomonas aeruginosa *1; PA 2: *P. aeruginosa *2; ST: *Salmonella typhi*; STM 1: *Salmonella typhimurium *1; STM 2: *S. typhimurium *2; SF: *Shigella flexneri*

### Antibacterial activity of organic solvent extract of seeds

Organic extracts showed similar results as observed in case of aqueous extracts with some variations. The extracts prepared in hexane and acetone gave relatively better inhibitory zones ranging from 9–30 mm (Table 2). Despite similar sensitivity pattern exhibited by hexane and acetone extracts, the latter is the preferred choice because of its polar nature, volatility, miscibility with polar and non-polar solvents and relatively lower toxicity [25]. Sensitivity of *E. coli*, *S. aureus *and *P. aeruginosa *to acetone extract of *A. graveolens *is in line with an earlier study [14]. Hexane extracts of *F. vulgare *and *T. ammi *did not show any antibacterial activity while alcoholic extracts had shown lesser activity [4] as compared to the present study. The differences observed could be due to the filtration of the extracts, which might have led to removal of the antibacterial components. In another study, methanolic extract of *A. graveolens *has not been reported to possess any antibacterial activity [26] while its ethanolic extract in the present study showed reasonable antibacterial potential. Aqueous and organic extracts of *A. graveolens *have shown anti-ulcer activity against *Helicobacter pylori *[27], providing the basis for their use as traditional gastro protective agents.

**Table 2:** Antibacterial activity of organic solvent extract of seeds

Bacteria	Extracts/Zone of inhibition (in mm)											
	P 1^a^				P 2^a^				P 3^a^			
	I^a^	II^a^	III^a^	IV^a^	I^a^	II^a^	III^a^	IV^a^	I^a^	II^a^	III^a^	IV^a^
EF	27	28	26	20	28	26	29	21	27	24	28	21
SA	30	27	30	23	29	23	28	21	28	26	30	25
EC	24	19	24.5	15	22	18	22	15	24	19	23	17
KP 1	-	-	-	-	-	-	-	-	14	11	13	12
KP 2	11	11	11	11	-	-	-	-	13	10	12	11
PA 1	14	12	12	12	9	-	9	-	13	12	15	12
PA 2	24	16	24	14	25	19	25	13	24	21	25	18
ST	18	15	18	-	18	14	20	13	19	17	18	15
STM 1	15	13	15	-	13	12	13	10	14	11	14	12
STM 2	23	22	26	21	24	21	26	16	24	18	24	20
SF	25	19	25	15	26	23	26	19	23	21	24	18
												
Mean ± SD	19.18 8.681	16.55 7.891	19.23 8.948	11.91 8.491	17.64 10.670	14.18 9.938	18.00 10.826	11.64 8.201	20.27 5.833	17.27 5.587	20.55 6.362	16.45 4.503

^a ^P1: *Anethum graveolens*; P2: *Foeniculum vulgare*; P3: *Trachyspermum ammi*

Organic extracts (I: Hexane; II: Ethyl acetate; III: Acetone; IV: ethanol)

-: no zone of inhibition

EF: *Enterococcus faecalis*; SA: *Staphylococcus aureus*; EC: *Escherichia coli*; KP 1: *Klebsiella pneumoniae* 1; KP 2: *K. pneumoniae* 2; PA 1: *Pseudomonas aeruginosa* 1; PA 2: *P. aeruginosa* 2; ST: *Salmonella typhi*; STM 1: *Salmonella typhimurium* 1; STM 2: *S. typhimurium* 2; SF: *Shigella flexneri*

The variations observed in the present study and earlier reports could be attributed to climatic and environmental conditions, strain differences, extraction protocol and the methods used to assess antimicrobial activity.

Gram positive bacteria were more sensitive than Gram negative and *P. aeruginosa *was the most sensitive among latter. The higher sensitivity of Gram-positive bacteria (*S. aureus *and *E. faecalis*) could be attributed to their outer peptidoglycan layer which is not an effective permeability barrier [28]. Gram-negative bacteria having an outer phospholipidic membrane carrying the structural lipopolysaccharide components make the cell wall impermeable to lipophilic solutes while porins constitute a selective barrier to hydrophilic solutes with an exclusion limit of 600 Da.

### Comparison of antibacterial activity of seed extracts with standard antibiotics

The different cultures responded to standard antibiotics and resulted in a variable inhibitory zone (9 to 38 mm) (Table 3). Aqueous and acetone seed extracts were better or equally effective against some of the bacteria as compared to standard antibiotics. Student's t-test showed statistically significant difference in antibacterial activity of seed extracts and antibiotics (P < 0.05) against *E. faecalis *and *P. aeruginosa *2 which however, were resistant to cefixime and chloramphenicol. Statistically, insignificant difference was observed among inhibitory activity of aqueous and acetone extracts. However, while comparing the antibacterial potential between aqueous and acetone extracts of each plant, acetone extract of *T. ammi *showed statistically significant activity in comparison to its aqueous extract (P < 0.05) while insignificant difference was observed for *A. graveolens *and *F. vulgare *extracts.

**Table 3:** Antibacterial activity of some standard antibiotics

Bacteria	Zone of inhibition (in mm)							
	Antibiotics (μg/disc)							
	A 10^a^	Cfx 5^a^	C 30^a^	Co 5^a^	G 10^a^	I 10^a^	Pt 10^a^	Tb 10^a^
EF	21	-	23	-	11	23	16	09
SA	30	21	22	21	17	39	33	18
EC	20	20	19	21	18	28	20	16
KP 1	-	22	23	24	14	21	18	17
KP 2	-	22	24	22	13	-	-	-
PA 1	-	-	16	-	17	21	17	19
PA 2	-	-	-	-	13	26	18	18
ST	10	17	19	16	15	21	17	13
STM 1	26	23	32	22	20	30	26	20
STM 2	-	20	23	18	21	24	17	17
SF	23	23	25	18	27	32	23	17
								
Mean	11.82	15.27	20.55	12.82	16.00	24.09	18.64	14.91
± SD	12.287	9.951	7.942	10.400	3.098	9.741	8.028	5.804

^a ^(μg/disc)

-: no zone of inhibition

A10: Ampicillin; Cfx5: Cefixime; C30: Chloramphenicol; Co25: Co-trimoxazole; G10: Gentamicin; I10: Imipenem; Pt10: Pipericillin/Tazobactam; Tb10: Tobramycin

EF: *Enterococcus faecalis*; SA: *Staphylococcus aureus*; EC: *Escherichia coli*; KP 1: *Klebsiella pneumoniae *1; KP 2: *K. pneumoniae *2; PA 1: *Pseudomonas aeruginosa *1; PA 2: *P. aeruginosa *2; ST: *Salmonella typhi*; STM 1: *Salmonella typhimurium *1; STM 2: *S. typhimurium *2; SF: *Shigella flexneri*

### Minimum inhibitory concentration

The strains which showed considerably good sensitivity to plant extracts were selected further to determine minimum inhibitory concentration (MIC) of aqueous and acetone extract of seeds. The MIC values were plant and strain dependent. The stronger extraction capacity of acetone could have yielded greater number of active constituents responsible for antibacterial activity. Better efficacy of acetone extracts was further supported by MIC studies (Table 4). The minimal inhibitory concentration for acetone and aqueous extracts ranged from 5–15 mg/ml and 20–80 mg/ml, respectively. The MIC value of aqueous extracts was 20 mg/ml for *A. graveolens *and *F. vulgare *while 60 mg/ml for *T. ammi*. Minimum inhibitory concentrations of essential oil of *F. vulgare *as worked out by Hammer *et al*. [29], supported our observation of greater sensitivity of *S. aureus *and even their MIC values ranging from 0.25% v/v (for *S. aureus*) to >2% v/v for other bacterial cultures are comparable to our studies. This highlights the equal effectiveness of aqueous and acetone extracts in comparison to essential oils.

**Table 4:** Minimum inhibitory concentration (mg/ml) of aqueous and acetone extracts of seeds

Bacteria	Plants					
	P 1^a^		P 2^a^		P 3^a^	
	Aq^a^	Ac^a^	Aq^a^	Ac^a^	Aq^a^	Ac^a^
Gram-positive						
*Enterococcus faecalis*	20	10	60	05	60	05
*Staphylococcus aureus*	20	05	60	05	70	05
Gram-negative						
*Escherichia coli*	40	10	60	10	80	10
*Pseudomonas aeruginosa *2	30	15	40	05	80	05
*Salmonella typhi*	30	15	30	05	80	10
*Salmonella typhimurium *2	20	05	20	05	80	05
*Shigella flexneri*	50	10	20	10	80	05

^a ^P1: *Anethum graveolens*; P2: *Foeniculum vulgare*; P3: *Trachyspermum ammi*

Aq: (Aqueous extract); Ac: (Acetone extract)

### Bactericidal activity using viable cell count studies

**Figure 1:**
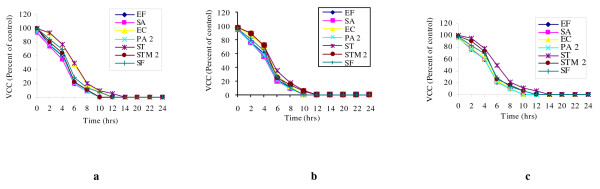
**Viable cell count (VCC) of different bacteria with hot water extract**. Viable cell count (VCC) of different bacteria with hot water extract of *Anethum graveolens *(a), *Foeniculum vulgare *(b) and *Trachyspermum ammi *(c).

The results obtained by viable cell count, lent further importance to the study and supported the data obtained by ADA and MIC. Hot water extracts (200 mg/ml) of all the three plant seeds led to complete killing of bacteria mainly within a time span of 10 h (Figure 1a–c). Their bactericidal nature was further confirmed as no re-growth occurred even after 24 h of incubation. *A. graveolens*, *F. vulgare *and *T. ammi *showed 90–92% killing of *S. aureus *after 8 h of incubation. *S. typhi*, which was least sensitive to aqueous extracts, took the longest time for complete inhibition.

### Qualitative and quantitative analysis of seeds for their phytoconstituents

Qualitative phytochemical analysis showed the presence of alkaloids, flavonoids, tannins, saponins and cardiac glycosides and the data for their quantitative determination has been presented (Table 5). Presence of tested secondary metabolites in the seeds of *A. graveolens *is in line with earlier reports [30,31]. The phytoconstituents detected in the plant materials could be responsible for their antimicrobial activity though their exact mode of action is poorly understood.

**Table 5:** Quantitative (percent) phytochemical evaluation of seeds of different plants

Plants	Alkaloids	Flavonoids	Tannins	Saponins	Cardiac Glycosides
*A. graveolens*	2.8 ± 0.10	11.05 ± 0.07	19.71 ± 0.28	0.55 ± 0.04	ND
*F. vulgare*	2.8 ± 0.17	15.06 ± 0.12	27.77 ± 0.10	0.55 ± 0.03	ND
*T. ammi*	4.23 ± 0.21	8.58 ± 0.19	22.77 ± 0.13	0.71 ± 0.09	ND

### Thin layer chromatography

**Figure 2:**
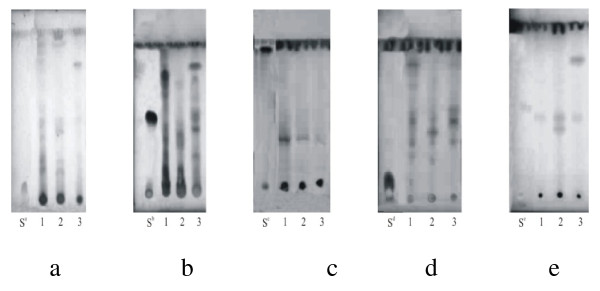
**TLC of *Anethum graveolens*, *Foeniculum vulgare *and *Trachyspermum ammi *extracts**. TLC of *Anethum graveolens *(1), *Foeniculum vulgare *(2) and *Trachyspermum ammi *(3) extracts. a: Alkaloids, b: Flavonoids; c: Tannins; d: Saponins; e: Cardiac glycosides. S: Standards (S^a^: Atropine, S^b^: Rutin, S^c^: Catechin, S^d^: Glycyrrhizic acid, S^e^: Lanatoside C).

The presence of phytoconstituents was further confirmed by thin layer chromatography and their R_f _values have been presented (Table 6, Figure 2a–e). The components were best resolved in screening system ethyl acetate/methanol/water (100:13.5:10), while atropine and glycyrrhizic acid could not be resolved in this system.

**Table 6:** R_f _values of different phytoconstituents

Plants	Alkaloids		Flavonoids		Tannins		Saponins		Cardiac glycosides	
	a	b	a	b	a	b	a	b	a	b
*A. graveolens*	0.226	0.186, 0.226, 0.426, 0.88	0.243, 0.328, 0.414, 0.757, 0.786	0.243, 0.328, 0.414, 0.757, 0.786	0.296	0.296	0.343, 0.390, 0.484	0.343, 0.390, 0.484, 0.734, 0.765, 0.812	-	0.441
*F. vulgare*	0.226	0.186, 0.226, 0.386	0.157, 0.286, 0.371	0.157, 0.286, 0.371, 0.657	0.312	0.312	0.328, 0.375, 0.484, 0.562	0.328, 0.375, 0.484, 0.562	-	0.382, 0.441
*T. ammi*	0.773	0.773	0.157, 0.243, 0.371, 0.457, 0.686, 0.8	0.157, 0.243, 0.371, 0.457, 0.586, 0.686, 0.8	0.296	0.296	0.375, 0.484, 0.546	0.375, 0.484, 0.546, 0.828	0.794	0.441, 0.794
Standard*	-	0.106		0.5, 0.557	0.92, 0.968	0.92, 0.968	-	0.093	-	0.514

a: Visible; b: UV 254 nm

*: Atropine (Alkaloid); Rutin (Flavonoid); Catechin (Tannin); Glycyrrhizic acid (Saponins); Lanatoside C (Cardiac glycoside)

### Antibacterial activity of isolated phytoconstituents

Isolated groups of compounds demonstrated their antibacterial effect though to a lesser extent (Table 7). *E. faecalis*, *E. coli*, *K. pneumoniae *and *P. aeruginosa *1 did not show any sensitivity. *S. aureus *was the most sensitive organism followed by *S. flexneri *and *P. aeruginosa*. Purified alkaloids as well as their synthetic derivatives are used as medicinal agents for their various biological effects such as analgesic, antispasmodic and bactericidal [9]. Flavonoids have also been reported to possess anti-bacterial activity, which could be attributed to their ability to form complex with extracellular, soluble proteins and bacterial cell walls [32]. However, in the present study qualitatively isolated alkaloids and flavonoids did not show any antibacterial activity as revealed by disc diffusion assay except for the sensitivity (6 mm) shown by *S. flexneri*. The difference observed in the inhibitory effect shown by alkaloids and flavonoids could be due to the variations in the methods used to assess their activity. Plant tannins, another class of polyphenolic compounds, results in their antimicrobial action by precipitating microbial protein [33] and this potency is governed by their concentration in the plants. In the present study, tannins showed inhibitory activity only against *P. aeruginosa *and *S. flexneri*. Mainly the antibacterial activity was shown by saponins, a special class of glycosides; though their concentration is much lower than that of flavonoids as revealed by quantitative analysis. Antibacteial activity shown by saponins against *S. aureus *is in consonance with an earlier study [34]. The present study revealed that the crude extracts contain a number of phytoconstituents whose isolation and purification may yield significant novel antimicrobial agents.

**Table 7:** Antibacterial activity of isolated group of phytoconstituents

Plants	Phytoconstituents	Bacteria Zone of inhibition (in mm)			
		SA	PA2	STM1	SF
*Anethum graveolens*	Alk	-	-	-	5.0
	Fl	-	-	-	-
	Tn	-	-	5.0	-
	Sp	9	6.5	4.8	7.5
	Cg	-	5.0	-	-
*Foeniculum vulgare*	Alk	-	-	-	6.0
	Fl	-	-	-	-
	Tn	-	6.0	5.0	6.75
	Sp	10	-	5.0	9.0
	Cg	-	-	-	-
*Trachyspermum ammi*	Alk	-	-	-	-
	Fl	-	-	-	-
	Tn	-	-	5.0	6
	Sp	11	-	5.0	8.5
	Cg	-	6.2	5.2	-

-: no zone of inhibition

SA: *Staphylococcus aureus*; PA2: *Pseudomonas aeruginosa*; STM1: *Salmonella typhimurium *1; SF: *Shigella flexneri*

Alk: Alkaloids; Fl: Flavonoid; Tn: Tannins; Sp: Saponins; Cg: Cardiac glycosides

The present work on three traditional medicinal plants showed their potential against the causative agents of nosocomial infections, *P. aeruginosa*, in particular; and important pathogens associated with various gastrointestinal disorders leading to indigestion, dysentery, and diarrhoea. Unfortunately, resistance to available antibiotics is on the rise and there are a limited number of antipseudomonal agents with reliable activity. Thus, the antibacterial activities of medicinal plants reported in the present study are noteworthy considering the importance of such microorganisms.

## Conclusion

In conclusion, seeds from all the three plants possessed equally good inhibitory activity against the tested bacteria. Aqueous as well as acetone extracts of seeds showed almost comparable antibacterial activity, which support their traditional use against infectious diseases. The presence of most general phytochemicals might be responsible for their therapeutic effects. It further reflects a hope for the development of many more novel chemotherapeutic agents or templates from such plants which in future may serve for the production of synthetically improved therapeutic agents.

## Competing interests

The authors declare that they have no competing interests.

## Authors' contributions

GJA carried out the experimentation as part of Ph.D. degree and drafted this manuscript. All this work was carried out under the supervision of DSA. Both the authors have read and approved the final manuscript.

## Pre-publication history

The pre-publication history for this paper can be accessed here:



## References

[B1] Cowan MM (1999). Plant products as antimicrobial agents. Clin Microbiol Rev.

[B2] Rios JL, Recio MC (2005). Medicinal plants and antimicrobial activity. J Ethnopharmacol.

[B3] Chopra I, Hodgson J, Metcalf B, Poste G (1997). The search for antibacterial agents effective against bacteria resistant to multiple antibiotics. Antimicrob Agents Chemother.

[B4] Ahmad I, Mehmood J, Mohammad F (1998). Screening of some Indian medicinal plants for their antimicrobial properties. J Ethnopharmacol.

[B5] Arora DS, Kaur J (1999). Antimicrobial activity of spices. Int J Antimicrob Agents.

[B6] Arora DS, Kaur GJ, Kaur H (2009). Antibacterial activity of tea and coffee: their extracts and preparations. Int J Food Properties.

[B7] Rojas JJ, Ochoa VJ, Ocampo SA, Munoz JF (2006). Screening for antimicrobial activity of ten medicinal plants used in Colombian folkloric medicine: A possible alternative in the treatment of non-nosocomial infections. BMC Complement Altern Med.

[B8] Iwu MW, Duncan AR, Okunjii CO, Janick J (1999). New antimicrobials of plant origin. Perspectives on new crops and new uses.

[B9] Evans WC (2002). Trease and Evans pharmacognosy.

[B10] Syed M, Sabir AW, Chaudhary FM, Bhatty MK (1986). Antimicrobial activity of essential oils of umbelliferae part II-*Trachyspermum ammi*, *Daucus carota*, *Anethum graveolens *and *Apium graveolens*. Pak J Sci Indigenous Res.

[B11] Ruberto G, Baratta MT, Deans SG, Dorman HJD (2000). Antioxidant and antimicrobial activity of *Foeniculum vulgare *and *Crithmum maritimum *essential oils. Planta Medica.

[B12] Singh G, Kapoor IP, Pandey SK, Singh UK, Singh RK (2002). Studies on essential oils: part 10; antibacterial activity of volatile oils of some spices. Phytother Res.

[B13] Lo-Cantore P, Iacobellis NS, De-Marco A, Capasso F, Senatore F (2004). Antibacterial activity of *Coriander sativum *L. and *Foeniculum vulgare *Miller Var. vulgare (Miller) essential oils. J Agri Food Chem.

[B14] Singh G, Maurya S, De-Lampasona MP, Catalan C (2005). Chemical constituents, antimicrobial investigations and antioxidant potentials of *Anethum graveolens *L. essential oil and acetone extract: Part 52. J Food Sci.

[B15] Arora DS, Kaur GJ (2007). Antibacterial activity of some Indian medicinal plants. J Nat Med.

[B16] Bauer AW, Kirby WMM, Sherris JC, Turck M (1966). Antibiotic susceptibility testing by a standardized single disk method. Am J Clin Pathol.

[B17] Mahajan V (1992). Comparative evaluation of sensitivity of human pathogenic bacteria to tea, coffee and antibiotics. PhD thesis.

[B18] Toda M, Okubo S, Hiyoshi R, Shimamura T (1989). The bactericidal activity of tea and coffee. Lett Appl Microbiol.

[B19] Harborne JB (2005). Phytochemical methods – A guide to modern techniques of plant analysis.

[B20] Wagner H, Bladt S (2004). Plant drug analysis-A thin layer chromatography atlas.

[B21] Bohm BA, Koupai-Abyazani MR (1994). Flavonoids and condensed tannins from leaves of Hawaiian *Vaccinium vaticulatum *and *V calycinium*. Pacific Sci.

[B22] Edeoga HO, Okwu DE, Mbaebie BO (2005). Phytochemical constituents of some Nigerian medicinal plants. Afr J Biotechnol.

[B23] Obadoni BO, Ochuko PO (2001). Phytochemical studies and comparative efficacy of the crude extracts of some haemostatic plants in Edo and Delta States of Nigeria. Global J Pure Appl Sci.

[B24] Van-Burden TP, Robinson WC (1981). Formation of complexes between protein and tannin acid. J Agri Food Chem.

[B25] Eloff JN (1998). Which extractant should be used for the screening and isolation of antimicrobial components from plants?. J Ethnopharmacol.

[B26] Bazzaz BS, Haririzadeh G (2003). Screening of Iranian plants for antimicrobial activity. Pharm Biol.

[B27] Rifat-uz-Zaman, Akhtar MS, Khan MS (2006). *In vitro *antibacterial screening of *Anethum graveolens *L. Fruit, *Cichorium intybus *L. leaf, *Plantago ovata *L. seed husk and *Polygonum viviparum *L. root extracts against *Helicobacter pylori*. Int J Pharmacol.

[B28] Nostro A, Germano MP, Angelo VD, Marino A, Cannatelli MA (2000). Extraction methods and bioautography for evaluation of medicinal plant antimicrobial activity. Lett Appl Microbiol.

[B29] Hammer KA, Carson CF, Riley TV (1999). Antimicrobial activity of essential oils and other plant extracts. J Appl Microbiol.

[B30] Mahran GH, Kardy HA, Isaac ZG, Thabet CK, Al-Azizi MM, El-Olemy MM (1992). Investigation of diuretic drug plants. 1. Phytochemical screening and pharmacological evaluation of *Anethum graveolens *L., *Apium graveolens *L., *Daucus carota *L. and *Eruca sativa *Mill. Phytother Res.

[B31] Bazzaz BS, Haririzadeh G, Imami SA, Rashed MH (1997). Survey of Iranian plants for alkaloids, flavonoids, saponins and tannins (Khorasan Province). Int J Pharmacog.

[B32] Tsuchiya H, Sato M, Miyazaki T, Fuziwara S, Tanigaki S, Ohyama M, Tanaka T, Iinuma M (1996). Comparative study on the antibacterial activity of phytochemical flavonones against methicillin-resistant *Staphylococcus aureus*. J Ethnopharmacol.

[B33] Scalbert A (1991). Antimicrobial properties of tannins. Photochem.

[B34] Soetan KO, Oyekunle MA, Aiyelaagbe OO, Fafunso MA (2006). Evaluation of the antimicrobial activity of saponins extract of *Sorghum bicolor *L. Moench. Afr J Biotechnol.

